# A picture is worth a thousand words: advancing the use of visualization tools in implementation science through process mapping and matrix heat mapping

**DOI:** 10.1186/s43058-023-00424-4

**Published:** 2023-04-25

**Authors:** Zachary M. Salvati, Alanna Kulchak Rahm, Marc S. Williams, Ilene Ladd, Victoria Schlieder, Jamie Atondo, Jennifer L. Schneider, Mara M. Epstein, Christine Y. Lu, Pamala A. Pawloski, Ravi N. Sharaf, Su-Ying Liang, Andrea N. Burnett-Hartman, Jessica Ezzell Hunter, Jasmine Burton-Akright, Deborah Cragun

**Affiliations:** 1 Geisinger Department of Genomic Health, 100 N. Academy Ave, Danville, PA 17822 USA; 2 Center for Health Research, Kaiser Permanente Northwest, 3800 N. Interstate Ave, Portland, OR 97202 USA; 3 Department of Medicine and the Meyers Primary Care Institute, University of Massachusetts Medical School, 365 Plantation St. Biotech 1, Suite 100, Worcester, MA 01605 USA; 4 Department of Population Medicine, Harvard Medical School and Harvard Pilgrim Health Care Institute, Boston, USA; 5 HealthPartners Institute, Bloomington, MN USA; 6 Division of Gastroenterology, Department of Medicine, Department of Healthcare Policy and Research, Weill Cornell Medicine, New York, NY USA; 7 Palo Alto Medical Foundation Research Institute, 795 El Camino Real, Palo Alto, CA 94301 USA; 8 Kaiser Permanente Colorado, Institute for Health Research, 2550 S. Parker Rd., Ste 200, Aurora, CO 80014 USA; 9 RTI International, 3040 East Cornwallis Road, P.O. Box 12194, Research Triangle Park, NC 27709-2194 USA; 10 University of South Florida, 3720 Spectrum Blvd, Suite 304, Tampa, FL 33612 USA

**Keywords:** Implementation, Optimization, Consolidated Framework for Implementation Research (CFIR), Process mapping, Matrix heat mapping, Data visualization, Cancer, Lynch syndrome, Tumor screening

## Abstract

**Background:**

Identifying key determinants is crucial for improving program implementation and achieving long-term sustainment within healthcare organizations. Organizational-level complexity and heterogeneity across multiple stakeholders can complicate our understanding of program implementation. We describe two data visualization methods used to operationalize implementation success and to consolidate and select implementation factors for further analysis.

**Methods:**

We used a combination of process mapping and matrix heat mapping to systematically synthesize and visualize qualitative data from 66 stakeholder interviews across nine healthcare organizations, to characterize universal tumor screening programs of all newly diagnosed colorectal and endometrial cancers and understand the influence of contextual factors on implementation. We constructed visual representations of protocols to compare processes and score process optimization components. We also used color-coded matrices to systematically code, summarize, and consolidate contextual data using factors from the Consolidated Framework for Implementation Research (CFIR). Combined scores were visualized in a final data matrix heat map.

**Results:**

Nineteen process maps were created to visually represent each protocol. Process maps identified the following gaps and inefficiencies: inconsistent execution of the protocol, no routine reflex testing, inconsistent referrals after a positive screen, no evidence of data tracking, and a lack of quality assurance measures. These barriers in patient care helped us define five process optimization components and used these to quantify program optimization on a scale from 0 (no program) to 5 (optimized), representing the degree to which a program is implemented and optimally maintained. Combined scores within the final data matrix heat map revealed patterns of contextual factors across optimized programs, non-optimized programs, and organizations with no program.

**Conclusions:**

Process mapping provided an efficient method to visually compare processes including patient flow, provider interactions, and process gaps and inefficiencies across sites, thereby measuring implementation success via optimization scores. Matrix heat mapping proved useful for data visualization and consolidation, resulting in a summary matrix for cross-site comparisons and selection of relevant CFIR factors. Combining these tools enabled a systematic and transparent approach to understanding complex organizational heterogeneity prior to formal coincidence analysis, introducing a stepwise approach to data consolidation and factor selection.

**Supplementary Information:**

The online version contains supplementary material available at 10.1186/s43058-023-00424-4.

Contributions to the literature

- Organizational complexity can be difficult to capture and analyze using existing implementation science approaches. Using the example multi-site study, we advance novel data visualization methodologies to systematically make comparisons and identify possible patterns, without losing the inherent rich nature of complex data.
- The combined data visualization methodologies of process mapping and data matrix heat mapping can help identify and/or operationalize key implementation outcomes and contextual factors.
- Combining these methods provides a novel stepwise approach to complex organizational data consolidation and factor selection for additional evaluation through comparative methods such as coincidence analysis, expanding the methods of implementation science.

## Background

Identification of common patterns across organizations can improve understanding of complex, interactive factors that facilitate and inhibit implementation processes and outcomes. However, studying the implementation and long-term sustainment of complex interventions involving multidisciplinary teams across organizations can be challenging. Examples of challenges include summarizing vast amounts of data from multiple stakeholders, reconciling inconsistencies, and identifying patterns across programs [1, 2]. Data visualization methodologies can assist with analysis and data presentation when comparing complex systems across organizations. Data visualization methods such as service blueprinting, journey mapping, and ecomapping have all been used in service design and health services research [3–5]. However, these methods focus primarily on “end-user” perspectives and illustrating care networks rather than highlighting organizational processes.

Process mapping, heat mapping, and data matrices are data visualization techniques that can help to summarize complex processes or multi-level data. A process map is a diagram or flow chart representing a sequence of actions to assist stakeholders in visualizing a given process or workflow [6]. Process mapping has been previously applied in health services research [7], quality improvement (QI), and quality assurance (QA) initiatives, but has not been widely used for contrasting processes/procedures across multiple sites in implementation research. Heat mapping is a data visualization technique that uses color to show magnitude differences within multiple conceptual ideas or data points. Heat maps are widely used in bioinformatics to visualize large gene expression data sets but have been underutilized in other areas [8, 9], including implementation science. Lastly, data matrices have been used to organize data and analyze complexities across multiple stakeholders and organizations. Constructing data matrices using the “Framework method” [10] helps summarize qualitative data from each stakeholder in a separate row of a spreadsheet and organizes data by themes within columns. The “matrixed multiple case study approach” recently described by Kim et al. [2] documents and compares multiple data sources both within and across organizational units to understand factors associated with program implementation. This method helped organize complex data into sortable matrices for cross-case comparisons, and researchers designated a single column to assign values for impact factors on program implementation. In their study, values were defined as “enabling,” “hindering,” or “neutral,” and success was defined based on implementation outcomes from Proctor et al. [11] Another example of using data matrices in implementation science is the “rapid-cycle evaluation approach to improving implementation” [12] where matrices helped organize data and support a cross-case analysis of patterns with barriers and facilitators. Each of these three approaches has been used to organize and analyze complex data. Although these have been widely used in other types of research, their use in implementation science is limited. We describe how we combined and built upon these methods with color-coding to aid in both data visualization and further consolidation of data, a method we call data matrix heat mapping.

We provide an example to demonstrate how process mapping and data matrix heat mapping can be used together to facilitate cross-case comparisons of factors that may impact implementation of a complex intervention or program at the organizational level. As part of the Implementing Universal Lynch Syndrome screening study (IMPULSS) [13], we refined and applied these methods to compare intervention protocols and processes across sites and identify factors relevant for program implementation and optimization. This methods manuscript details how the novel combination of process mapping and matrix heat mapping helped achieve our study objectives. The step-by-step methods we describe could be applied and/or modified for use in other implementation science research.

## Methods

### Study setting

The goal of IMPULSS is to characterize variation in healthcare system processes used to screen individuals with endometrial and colorectal tumors to identify Lynch syndrome (LS), a hereditary cancer predisposition condition [14]. This screening process, commonly referred to as universal tumor screening (UTS), is a multi-step complex intervention that requires coordination across multiple departments and stakeholders within a health system. Ultimately, the goal of a UTS program is to identify more patients with LS who will benefit from condition-specific medical care by identifying and/or preventing cancers early, thereby improving patient outcomes. UTS for both colorectal and endometrial cancers has been found to be both feasible and beneficial [15, 16], with support from several professional organizations including the National Comprehensive Cancer Network (NCCN), the American College of Obstetrics and Gynecology (ACOG), the American Society for Clinical Pathology (ASCP), and the Evaluation of Genomic Applications in Practice and Prevention (EGAPP) working group [17–21]. Despite strong evidence of patient benefit and over a decade of support for UTS, a standard approach for implementing a program is lacking, and variability has been identified within UTS protocols across healthcare organizations [22, 23]. Hence, key IMPULSS study objectives were to compare variation in site-specific protocols, determine contextual factors that might make a difference in organizational decision-making to implement UTS programs, and identify components related to program optimization.

The IMPULSS study design is described in detail elsewhere [13]. Briefly, nine healthcare systems participating in IMPULSS were chosen for their variability in having or not having a UTS program. To understand program variation and barriers and facilitators to UTS program existence or implementation, data collection consisted of four researchers interviewing 66 stakeholders across the healthcare systems. A semi-structured interview guide was developed and employed based on the Consolidated Framework for Implementation Research (CFIR) and contained several additional questions to elucidate processes of the tumor screening protocols at each organization (see Fig. 1 of supplemental materials for example). Interviews were conducted by ZS, IL, VS, and JA by telephone or Microsoft Teams, were recorded, transcribed verbatim, and coded as outlined below in step 1 of both process mapping and data matrix heat mapping. CFIR constructs, combined constructs, and domains [24] are hereafter referred to as “factors” to simplify terminology. The process mapping and matrix heat mapping methods described below are all constructed from interview data.

### Process mapping methodology and output

**Fig. 1:**
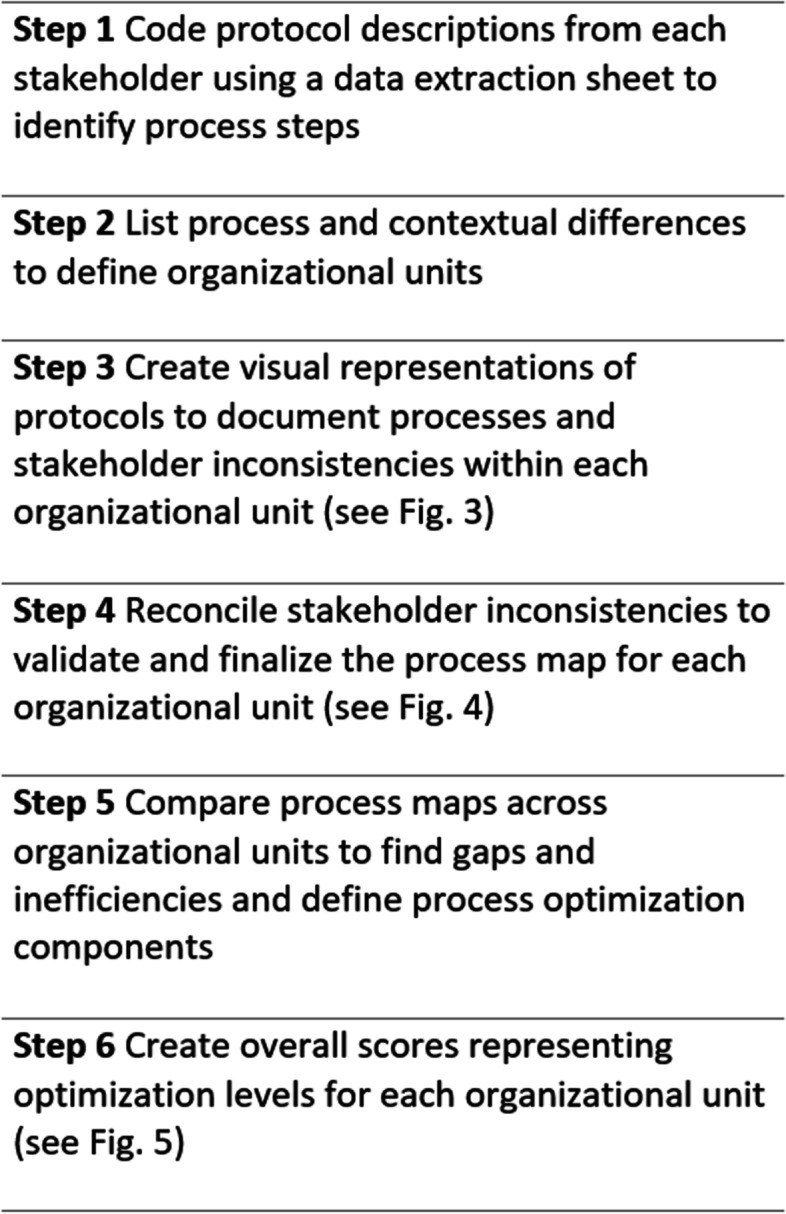
Process mapping methodology to quantify level of implementation success (i.e., optimization scores)

The goal of process mapping was to (1) visually represent and easily compare each organization’s UTS protocol, (2) help visually pinpoint barriers to patient care that may affect process optimization, and (3) identify components to help quantify implementation optimization levels. Process mapping was led by ZS, with consultation from study team members, and guided by literature [7], including a previous study conducting cross-case comparison using data visualization of UTS protocols [25] and a study looking at factors associated with patient follow-through after a positive screen [22]. We used coded interviews combined with prior literature [22, 25] to conduct process mapping. Our approach to process mapping was highly iterative and consisted of six main steps (see Fig. 1) outlined below.

#### Step 1: code protocol descriptions from each stakeholder using a data extraction sheet to identify process steps

First, ZS and JA, along with a research assistant, reviewed two stakeholder transcripts to create an initial data extraction sheet of key components and procedures (i.e., process steps) that make up UTS protocols informed by prior research [22, 25] and NCCN guidelines [17]. All stakeholder transcripts from a single organization were combined and analyzed together. Discussions among authors resulted in clarifications to the extraction sheet as questions arose while completing data extraction and coding of the remaining transcripts. During this process, commonalities and differences in procedures were identified across and within organizations.

#### Step 2: list process and contextual differences to define organizational units

During step 1, conflicting information between some stakeholders within the same healthcare organization prompted us to list process inconsistences and contextual differences in both structural characteristics and geographic location. ZS, AKR, and DC listed and sorted stakeholders by these differences (see Fig. 2 of supplemental materials for example), occasionally identifying different protocols within a single healthcare organization. Organizational units were defined as unique sites for analysis, each with their own protocol for identifying patients with LS.

#### Step 3: create visual representations of protocols to document processes and stakeholder inconsistencies within each organizational unit

ZS reviewed data from Steps 1 and 2 to create a process map of each organization’s approach to identifying LS (see Fig. 3 of supplemental materials for example). These preliminary process maps highlighted discrepancies across stakeholders and helped lead to the creation of additional process maps if multiple organizational units were identified during the prior step or current step.

#### Step 4: reconcile stakeholder inconsistencies to validate and finalize the process map for each organizational unit

ZS and DC conducted formal reconciliation and validation of the process map for each organizational unit. Minor discrepancies that remained after creating separate maps were resolved by weighing perspectives differently depending on the roles and responsibilities of the interviewed participant. Greater emphasis was given to the perspectives of primary stakeholders, defined as a provider stakeholder working directly with implementation and/or maintenance within the UTS protocol. ZS and DC led member checking where process maps were shown to respective stakeholders. Member check meetings were used to share, confirm, reconcile, and clarify collected data and related interpretations among stakeholders. Feedback was used to adjust process maps as needed. The outcome of this step was a final process map for each of the 19 organizational units (see Fig. 4 of supplemental materials for example).

#### Step 5: compare process maps across organizational units to find gaps and inefficiencies and define process optimization components

Once each process map was finalized, we sought to find commonalities in process optimization components between organizational units. ZS, in consultation with AKR and DC, conducted a cross-case comparison by cross-referencing each process map with coded transcripts and highlighting similarities and differences between organizational units. This comparison via data visualization helped ZS, AKR, and DC find common points within the UTS protocols where tumor screening and/or genetics referrals were not completed. These patient care barriers were listed and defined as process gaps and inefficiencies that were used for optimization scoring in the next step (see Fig. 5 of supplemental materials).

#### Step 6: create overall scores representing optimization levels for each organizational unit

ZS, DC, and AKR settled on five components associated with an optimized UTS program to help create an optimization scoring system. These components were selected based on descriptions by interviewed participants when discussing process execution and program evaluation and informed by previous findings [22, 25]. Each organizational unit was coded as “1”, “0.5”, or “0” for each optimization component using the following definitions determined by the study team: A “1” indicated presence of the component, a “0” indicated absence, and “0.5” was typically assigned if the organizational unit previously had an optimization component, such as quality assurance, that they no longer conduct or is no longer present. Additionally, one organizational unit received a “0.5” because it remained unresolved due to stakeholder discrepancy. Upon totaling the optimization scale, sites that had implemented a UTS protocol could receive a maximum total score of 5 and those that had not implemented a protocol were assigned a total score of 0. During our member-checking process, we shared the optimization scores to verify their accuracy and “face validity” according to stakeholder experience. We incorporate these scores in a subsequent step within the data matrix heat mapping process described below (step 5).

### Data matrix heat mapping methodology and output

**Fig. 2:**
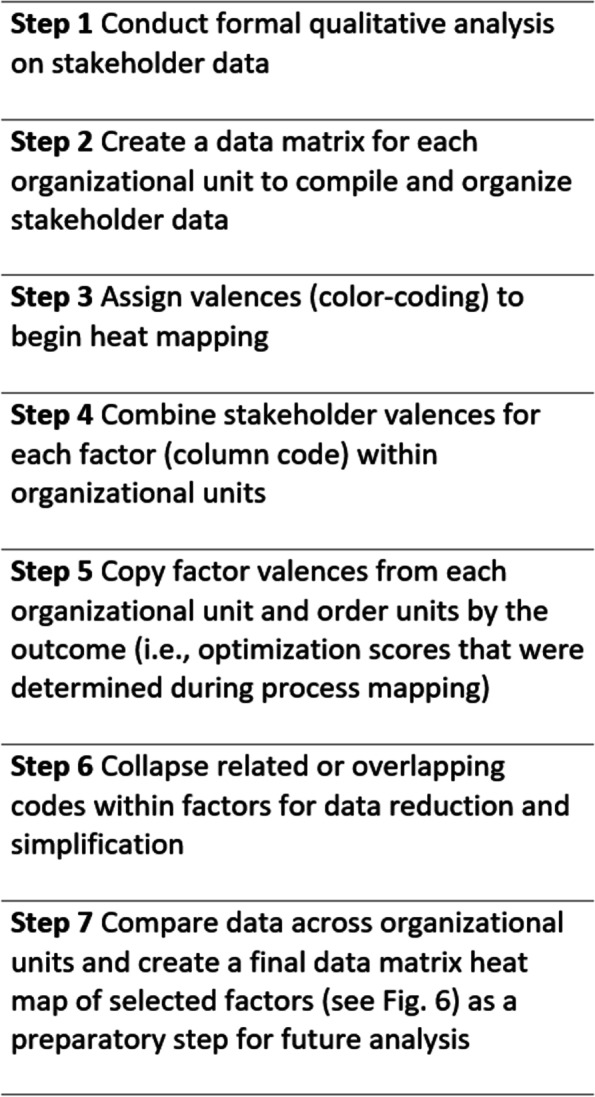
Data matrix heat mapping methodology to compile, organize, and consolidate complex data

Steps for data matrix heat mapping were developed concurrently as the study team operationalized the outcome using process mapping. The goal of matrix heat mapping was to consolidate, organize, and select relevant CFIR factors as a necessary, preparatory step for our planned future analysis. ZS and AKR have prior experience with using the Framework method [10] to organize interview data, and DC has prior experience using color-coding as a visual method to designate differences across cases. Additional inspiration for our approach came from the multiple matrixed case study approach [2] and prior studies that applied CFIR coding to qualitative data [24, 26, 27]. Data matrix heat mapping (illustrated in Figs. 7–13 of the supplementary materials) consisted of seven main steps (see Fig. 2) to compile and code contextual data within each organizational unit using multiple matrices that were ultimately combined into a single data matrix to compare contextual factors and outcomes across all units.

At various points throughout the data matrix heat mapping methodology, ZS and DC referred to transcripts and conducted member-checking with primary stakeholders and site PIs (AKR, JLS, MME, CYL, PAP, RNS, SYL, ANBH, and JEH) for verification and to evaluate the context in which quotations or summaries were extracted [28]. This type of iterative approach was applied to capture nuance within the rich data and to improve the rigor and accuracy of coding.

#### Step 1: conduct formal qualitative analysis on stakeholder data

A formal qualitative coding process guided by the CFIR framework was employed with interview transcripts by a two-person team of JBA and DC. First, JBA and DC used the CFIR codebook to code two interview transcripts. Throughout early coding, JBA and DC made modifications to the codebook with consultation from the study team to add inclusion and exclusion criteria specific for UTS implementation. Next, JBA continued coding all transcripts using RQDA qualitative software [29], with DC sorting codes by CFIR factor and reviewing text coded within each factor. Throughout coding, discrepancies were discussed and resolved by JBA and DC with input from additional study personnel as needed.

#### Step 2: create a data matrix for each organizational unit to compile and organize stakeholder data

DC and two research assistants summarized the coded data to create a data matrix for each organizational unit. To do this, CFIR factors were listed in columns and relevant quotes/summaries were included in the respective cells such that responses from each stakeholder interviewed were represented across a single row within the data matrix (i.e., spreadsheet) for their respective organizational unit.

#### Step 3: assign valences using color-coding to begin heat mapping

We applied a modified CFIR data coding approach to assigning valences that included the use of color-coding [26, 30]. Specifically, ZS and DC reviewed each data matrix and independently color-coded the summaries for each construct for each stakeholder according to the following valences: positive or presence of facilitator = blue, negative or presence of a barrier = red, mixed (i.e., both positive and negative) = purple and “neutral/non-salient” = gray. ZS and DC compared color coding and resolved most discrepancies through discussion and review of the original transcripts to help contextualize and ensure accuracy of summaries. In a few instances, discrepancies were resolved with input from additional study personnel.

#### Step 4: combine stakeholder valences for each factor (column code) within organizational units

ZS and DC applied rules for combining valences (see Fig. 10 of supplementary materials) from individual interviews within the same organizational unit to create a new row of summary valences for each organizational unit. For example, when one factor for a stakeholder had a “positive” valence and a different stakeholder had a “negative” valence, that factor became “mixed” for the organizational unit.

#### Step 5: copy factor valences from each organizational unit and order units by the outcome (i.e., optimization scores that were determined during process mapping)

Once the initial data matrices were completed, we combined summary valences from data matrix heat maps of each organizational unit into a single data matrix to compare contextual factors more easily across organizational units. DC copied all coded factors for each organizational unit into their respective CFIR domain to create a single data matrix containing the summary valences for all organizational units. One organizational unit was not included due to missing data, leaving 18 organizational units for analysis. Optimization scores were added to the matrix and organizational units were ordered from highest to lowest based on their optimization score. We then divided organizations into three groups based on natural breaks in the actual organizational optimization scores which grouped as follows: (1) score of “5″ = ”fully optimized,” (2) score of "0″ = “no program,” and (3) the remainder with scores ranging between “1″ to “3.5″ = ”non-optimized.”

#### Step 6: collapse related or overlapping codes within factors for data reduction and simplification

DC, with input from the research team, collapsed related CFIR factors. For example, “evidence strength and quality” and “relative advantage” were combined because they were often discussed together in stakeholder responses and usually had the same valence for each organizational unit. Specifically, the evidence that stakeholders often cited was how UTS did a better job at identifying LS compared to other approaches. As another example, most factors related to an organizational unit’s inner setting such as “networks and communications”, “culture”, “implementation climate”, and “readiness for implementation” were combined to create an overall inner setting valence. However, “structural characteristics” were not hypothesized to impact the outcome directly and were therefore not combined as part of overall setting (color-coding rules for collapsing factors can be seen in Fig. 12 within the supplementary materials for a visual representation).

#### Step 7: compare data across organizational units and create a final data matrix heat map of selected factors as a preparatory step for future analysis

A finalized and updated data matrix heat map with optimization scores from process mapping was reviewed to conduct cross-case comparisons and select CFIR factors. The color-coded visualization of the matrix heat map was used by the study team to hypothesize which factors may distinguish between optimized organizational units, non-optimized organizational units, and those without a UTS program. Factor selection was the final preparatory step before conducting coincidence analysis to formally test whether the hypothesized factors make a difference for the outcome as described in the initial IMPULSS study design [13].

## Results

### Process mapping results and interpretations

Process mapping revealed variation in protocols implemented to identify LS patients, both within and across organizations. UTS protocols included ten main steps which were partially impacted by differences in how institutions are structured and involvement of geographically distinct pathology groups.

**Fig. 3:**
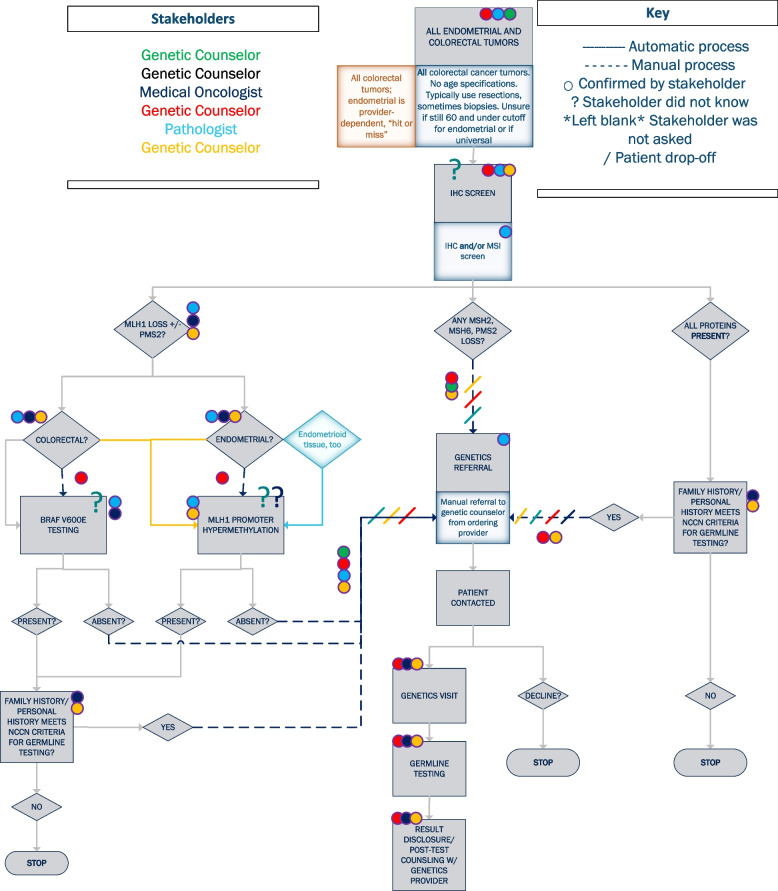
Section of the initial process map for organizational unit 1A. Key describes lines and symbols that were color-coded to represent varying stakeholder perspectives and to signify corroboration across stakeholders within an organization. Symbols were also used to identify and visually discern inconsistencies in stakeholder-reported interview data. Ten total stakeholders were interviewed from organization 1. Stakeholders not pictured were located at other organizational units (1B, 1C, and 1D)

**Fig. 4:**
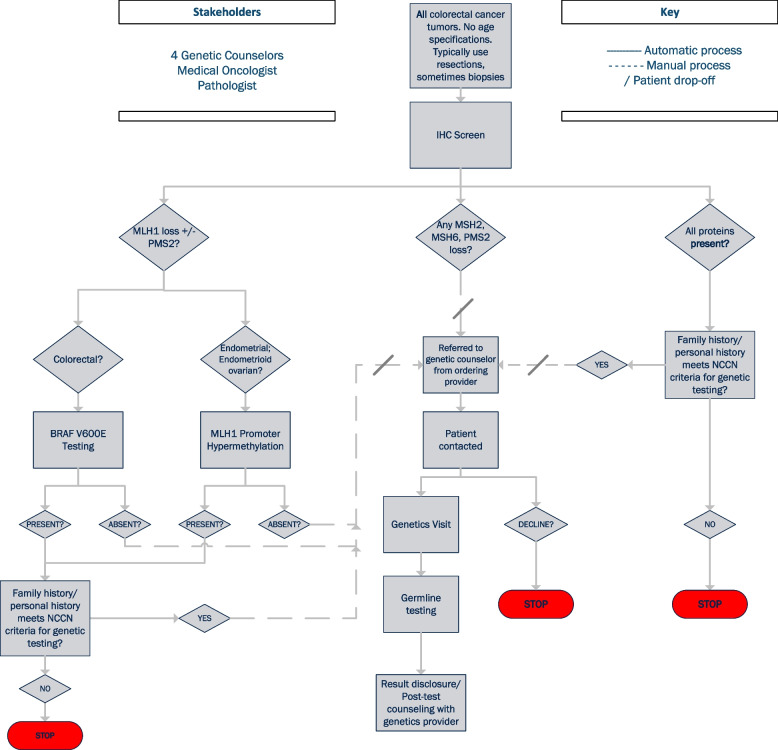
Section of the reconciled process map for organizational unit 1A. Completion of process mapping resulted in 19 reconciled process maps, one for each organizational unit

Most (67%) of the healthcare organizations we interviewed had a single protocol for identifying LS, but three of the nine were divided into multiple organizational units. These findings led to the creation of 19 finalized process maps, one for each organizational unit identified (see Figs. 3 and 4 for examples of initial and reconciled/finalized versions, respectively). This visual representation assisted with systematically comparing protocols and detecting process gaps and inefficiencies related to inconsistent execution of the tumor screening protocol and referrals, lack of process automation, and no evidence of data tracking or quality assurance. These gaps and inefficiencies were related to five analogous optimization components that were used to calculate the optimization score:

- Routine or consistent execution in conducting either immunohistochemistry (IHC) or microsatellite instability (MSI) testing. Note: Both methods are appropriate as an initial tumor screen to identify patients with LS [17] (IHC or MSI consistency)
- The use of reflex testing using *BRAF* V600E and/or *MLH1* promoter hypermethylation testing on a subset of “screen positive” tumors (*MLH1* absent) as recommended by the NCCN [17] (routine reflex)
- Consistency in referrals of patients with positive screens for germline genetic testing (referral consistency)
- Systematic results tracking (evidence of tracking)
- Ongoing quality assurance measures (quality assurance)

The first three components of the optimization score (IHC or MSI consistency, routine reflex, and referral consistency) correlate to the CFIR process factor of “executing.” The last two components (evidence of tracking and quality assurance measures) fall within the CFIR process factor of “reflecting and evaluating.”

**Fig. 5:**
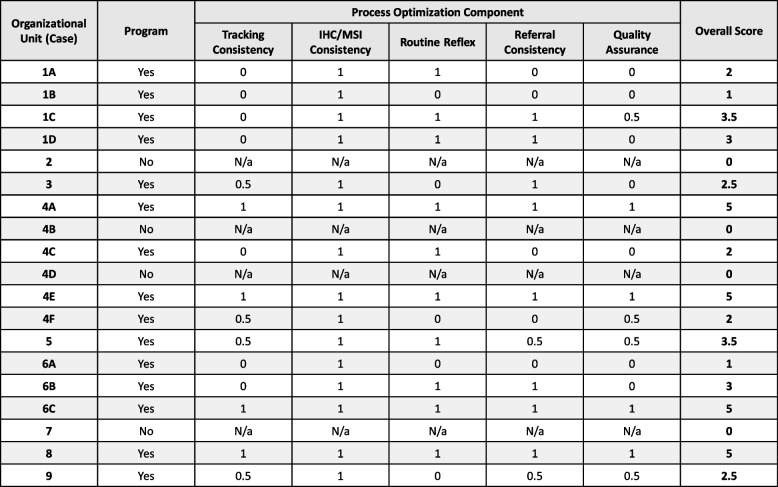
Matrix of UTS protocol optimization levels by organizational unit. Presence of process optimization component = “1;” historical presence without current presence of optimization component, or unresolved discrepancy between stakeholders = “0.5;” absence of process optimization component = “0.” Optimization components were not applicable (N/a) at organizational units without a program and those received an overall optimization score of “0”

Four organizational units scored a 5 out of 5, indicating full UTS program optimization. The remaining 11 organizational units with a UTS program were not optimized. Given that scores for non-optimized programs were all between 1 and 3.5, all organizational units would all have been categorized the same regardless of whether we used 0.5 in our coding system. A total of four organizational units had no program and were included in the study to understand reasons why healthcare systems may not have a UTS program. Organizational units had varied optimization scores, including sites under a single organizational umbrella. As illustrated in Fig. 5, organization 4 had six units ranging from a score of 0 to 5. Likewise, we saw varied UTS protocol execution at organization 6 with scores ranging from 1 to 5.

### Data matrix heat mapping results and interpretations

**Fig. 6:**
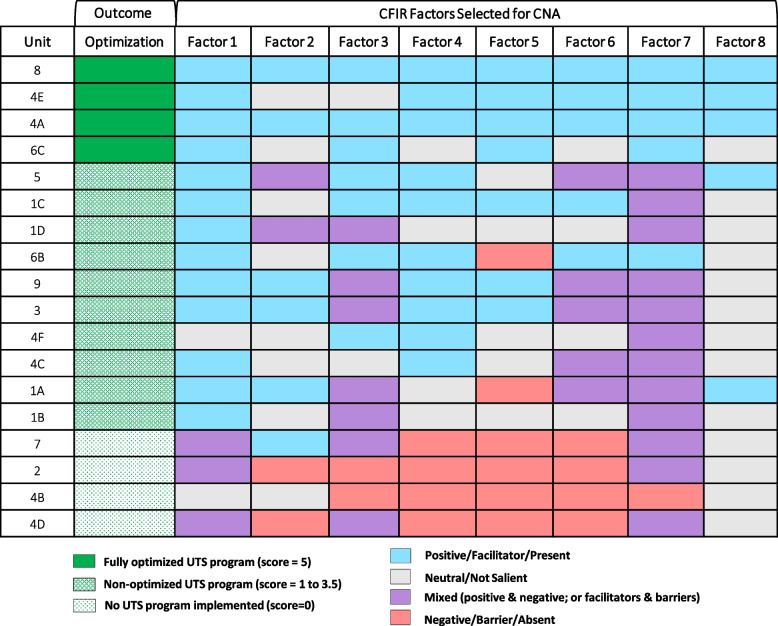
Final heat map showing the outcome and factors selected for coincidence analysis (CNA). Factor 1: evidence and relative advantage; Factor 2: cost; Factor 3: knowledge and attitudes of stakeholders; Factor 4: implementation champion; Factor 5: maintenance champion; Factor 6: planning and engaging stakeholders; Factor 7: inner setting (except structural); Factor 8: external networks (cosmopolitanism), peer pressure

The final heat map shown in Fig. 6 (also seen in Fig. 13 of supplemental materials) shows organizational units ordered based on their outcome scores from process mapping.

Each row represents a single organizational unit with the selected color-coded factors organized in columns. Factors were selected by the research team because they were hypothesized to influence UTS implementation and/or optimization based on experience and literature review [11, 12, 15, 22, 31, 32]. The final data matrix heat map shows more blue patterns (presence of factor/facilitator/positive influence) seen at fully optimized sites, and more purple (mixed perceptions) and red (absence of a factor/presence of a barrier/negative influence) patterns at non-optimized sites and sites without a program.

The positive blue coloring in evidence and relative advantage (Factor 1) (Fig. 6) shows that 13 of 14 sites with a program, regardless of optimization level, had stakeholders who saw clear benefits and strong evidence favoring UTS; in contrast, purple coloring shows mixed perceptions among stakeholders at three of the four organizational units with no program. Cost (Factor 2) was included in our final factor selection because it was identified as the primary concern at one organizational unit and other literature discusses its importance to implementation [11, 31, 32]. Knowledge and attitudes of stakeholders (Factor 3) was included given that it showed a greater presence of blue among units with higher optimization scores.

The following quote illustrates the potential impacts of both knowledge and attitudes of stakeholders (Factor 3) and inner setting (Factor 7), “I think [UTS] is the kind of thing that [provider stakeholders] would be interested in [positive attitudes], so it may just be a matter of distribution of resources or where it’s going to fall with other priorities [inner setting]” — Genetic counselor, Unit 1D. Four of the five fully optimized sites had a positive inner setting. In contrast, programs that were not fully optimized had a mixed inner setting. Implementation champion (Factor 4) was selected due to prior research supporting the key role implementation champions can play [33–35] as well as our observation that this came up multiple times in qualitative data as evidenced by the following quote: “Make sure you have the right people at the table at the right time. I think that’s key, making sure you have the right [team] and identifying a champion, somebody who is really going to champion what you’re doing and show people why [UTS] is important” — Tumor registrar, Unit 3.

Evidence of a maintenance champion (Factor 5) was present at all the optimized sites. Even though a maintenance champion was also present at some non-optimized sites, qualitative data support the importance of a maintenance champion who is often not the same person as the implementation champion: “One of our other genetic counselors is taking some ownership of [UTS] now, but she has more regular touch-ins with those pathologists. A specific representative from the department is often helpful” — Genetic counselor, Unit 8.

Planning and engaging stakeholders (Factor 6) is logically necessary for implementation and optimization of UTS programs. In fact, only sites without a UTS program are lacking planning and engaging.

Lastly, cosmopolitanism (i.e., external networks) and peer pressure (Factor 8) was included as the final factor since stakeholders from multiple organizational units described their importance: “We address our algorithm every other year to reach out to [a leading healthcare organization in UTS] and the researchers to see where things are at and do review of the literature to update and see if we’re in line with [peers at leading organizations]” — Nurse practitioner, Unit 4A; “We emailed friends at other institutions. We weren’t starting from scratch, and you don’t have to. Don’t reinvent the wheel, just figure out what’s going on out there and what can work best for your institution” — Genetic counselor, Unit 5.

## Discussion

In this methods manuscript, we described how two data visualization methods were useful in identifying and comparing factors that may be associated with the implementation and optimization of complex programs within healthcare organizations, specifically the implementation of UTS for LS. The first data visualization method, process mapping, used color-coded symbols to represent stakeholder descriptions of current UTS processes within their organization. Ecomapping [5] is a similar method that incorporates color-coded symbols to represent supportive care networks and individual stakeholder relationships, but process mapping helped to visualize and easily compare organizational processes. In our study, process mapping led to the identification and classification of distinct organizational units that we used to conduct cross-case comparisons, uncover similarities and differences across organizational units, and gain greater insight into organizational complexity impacting implementation variability.

Process mapping has been used previously for data visualization of UTS programs across multiple healthcare systems but was not used to document and reconcile stakeholder inconsistencies. Palter et al. [25] found one of the same components for the implementation optimization of UTS programs, namely ensuring the coordination of patient tracking to optimize the pathway for possible LS patients to meet with genetic counselors. The other key facilitators to successful implementation identified by Palter et al. were provider-stakeholder engagement and flexibility to tailor programs to relevant clinical sites. Our study expands on the findings of Palter et al. by identifying additional components for program optimization that we used to define implementation success and develop a scale measuring the degree to which a program has been optimally implemented and maintained.

Although the use of data matrices to make comparisons across sites is not new [2, 10] our study expanded its use by incorporating color-coded valences for factors believed to be important to implementation and optimization. We propose that this novel method of data matrix heat mapping offers a systematic approach to organize complex institutional data, assign valences, and conduct cross-site comparisons. One of the benefits of matrix heat mapping is that it allows researchers to see patterns within the final data matrix. Another benefit that matrix heat mapping shares with the multiple matrixed case study approach is that these approaches allow researchers to go back to the earlier data matrices to review additional details/summaries and easily gain a more nuanced or detailed understanding of each organizational unit. The earlier matrices can also be linked directly to supporting quotes. Furthermore, matrix heat mapping helped formalize a data consolidation technique for combining data from multiple stakeholders. Finally, data matrix heat mapping also provided a systematic approach to factor reduction, which is a necessary step before conducting other, more formal analyses such as coincidence analysis (CNA).

We plan to conduct CNA as the final analytic step in the IMPULSS study [13] to test whether and how the factors we selected using matrix heat mapping consistently make a difference for implementation and optimization. One advantage of CNA is its ability to identify whether the data support underlying causal complexity (e.g., more than one factor may be minimally necessary and sufficient for the outcome or multiple factors may form a causal chain that led to the outcome) [27, 36]. Currently, CNA is limited in the number of factors that can be successfully analyzed by the computer algorithm which builds models that fit the data and meet certain consistency and coverage thresholds. Others have used a data-driven methodology to select factors for CNA [37], and our matrix heat mapping approach provides another method to review and select factors for use in CNA.

## Limitations

This study and the data visualization approaches described here have several limitations. First, data collection was limited to current stakeholders at the organizations in the IMPULSS study, some of whom were newer to the organization and lacked historical information related to the initial UTS program implementation. This may limit the ability to fully assess whether and how implementation champions impacted UTS program implementation at those organizational units. Several prior studies [33–35] have identified implementation champions as one of the necessary components for success, and several participants in our study described the important role champions played in their program implementation. However, we had one organizational unit that clearly lacked an implementation champion but had a non-optimized program due to their partnership with an external hospital that initiated tumor screening.

An additional limitation is that participant roles in the process of identifying LS and the number of stakeholders across organizational units varied. The study team attempted to bridge gaps in knowledge that were not obtained in the initial interviews by conducting member-checking with participants and their colleagues, but there were some organizational units where member-checking was not logistically possible.

Furthermore, although attempts were made to identify multiple stakeholders, a few organizational units had only one stakeholder perspective represented, especially among organizations that we ultimately divided into multiple units. Consequently, one site with a non-optimized program was excluded from the data matrix heat mapping analysis because the sole stakeholder interview provided limited information. The variability in stakeholders interviewed may have impacted results because certain CFIR factors were identified to be more likely assigned a valence of “non-salient” (color-coded gray) at organizational units where only a single stakeholder was interviewed. Factors deemed “non-salient” should not make a difference for the outcome because we expect that relevant factors would be mentioned in stakeholder interviews upon probing. Nevertheless, in some instances, these “non-salient” factors may represent missing perspectives of other stakeholders. The presence of multiple gray “non-salient” factors served as a secondary impetus for factor consolidation, although the primary impetus of consolidation was to conduct the planned CNA. The use of our consolidation process may obscure some of the nuanced details. For example, by combining aspects of the inner setting, it is not clear which ones are most critical. However, one benefit of our approach is that it allowed us to review earlier data matrices and we discovered that the most salient inner setting factors differed. For several organizational units, it was the presence of strong networks and communications, but for others, it was primarily the presence of available resources.

Additionally, program optimization was defined, in part, based on current NCCN guidelines [17], and guidelines may change as evidence evolves and healthcare organizations choose other strategies for LS identification such as tumor sequencing [38, 39] or direct-to-germline [39, 40] sequencing. Future studies following the methodological steps we describe will need to review current literature to assess for updates in guidelines since the implementation of new procedures could alter how optimization is defined or which factors are related to implementation and optimization.

While we did not directly test this approach against other methods, we identified limitations in existing methods. Specifically, while working to identify process optimization components, data complexity necessitated a way to consolidate and visualize data to readily identify, document, and resolve discrepancies across multiple stakeholder perspectives. Furthermore, the process maps made member checking much easier because it provided a quick way to visualize all key steps or gaps in each organization’s UTS process. In addition, we found that using the multiple matrixed method described by Kim et al. [2] did not meet our needs to consolidate data and highlight patterns to ultimately select factors for further analysis. This was the impetus for developing the color coding and consolidation process steps of matrix heat mapping.

This is the first study to apply these two data visualization techniques in tandem. Therefore, future studies that incorporate process mapping and data matrix heat mapping are needed to sufficiently validate this approach. Nevertheless, this work demonstrated proof of concept for using this approach in implementation science research.

## Conclusion

We offer a detailed description of how two data visualization techniques were used to successfully organize and analyze data from an implementation study of a complex program (UTS) with multiple stakeholders and organizational units. Process mapping provided an efficient method to visualize protocols and operationalize the key optimization factors to create scores representing our implementation outcome. Matrix heat mapping using a priori CFIR factors helped identify local contextual factors impacting program implementation and optimization. Combining both the primary outcome (i.e., optimization scores from process mapping) and the summarized qualitative interview data formed the basis for our final “data matrix heat map.” These data visualization methods also helped consolidate factors that can be used for formal analysis using CNA to determine which of the factors differentiate between organizational units with an optimized program, a non-optimized program, or no program. These methodological approaches (or variations of them) may be useful for other implementation science studies aimed at understanding the impact of multiple organizational factors on implementation processes and outcomes.

## Supplementary Information

**Additional file 1:** **Fig. 1.** Data Extraction Guide for Universal Tumor Screening (UTS) Protocol Processes. **Fig 2.** Process and Contextual Differences within Organization 1. **Fig 3.** Initial Process Map for Organizational Unit 1A. **Fig 4.** Reconciled Process Map for Organizational Unit 1A. **Fig. 5.** Process Gap/Inefficiency to Optimization Component Conversion Table. **Fig. 6.** Matrix of UTS Protocol Optimization Levels by Organizational Unit. **Fig. 7.** Section of the CFIR Codebook Used for Qualitative Data Analysis. **Fig. 8.** Data Matrix of Factors Related to Intervention Characteristics from Organizational Unit 7. **Fig. 9.** Example of a Data Matrix Heat Map for Factors Related to Intervention Characteristics. **Fig. 10.** Example of Combined Codes for Factors Related to Intervention Characteristics. **Fig. 11.** Consolidated Data Matrix Heat Map for Characteristics of Intervention by Optimization Score. **Fig. 12.** Example of Collapsed Code ‘Evidence & Relative Advantage’ for all Organizational Units. **Fig. 13.** Final Heat Map of Factors Selected for Coincidence Analysis (CNA).

## Data Availability

All data generated or analyzed during this study are included in this published article and its supplementary information files.

## Abbreviations

NCI: National Cancer Institute
LS: Lynch syndrome
UTS: Universal tumor screening
dMMR: Mismatch repair deficiency
IHC: Immunohistochemistry
MSI: Microsatellite instability
NCCN: National Comprehensive Cancer Network
ACOG: American College of Obstetrics and Gynecology
ASCP: American Society for Clinical Pathology
EGAPP: Evaluation of Genomic Applications in Practice and Prevention
SGO: Society of Gynecologic Oncology
QI: Quality improvement
QA: Quality assurance
CFIR: Consolidated Framework for Implementation Research
CNA: Coincidence analysis
