# Supplementary material for: A picture is worth a thousand words: advancing the use of visualization tools in implementation science through process mapping and matrix heat mapping
Source: Implement Sci Commun. 2023 Apr 25;4:43. doi: 10.1186/s43058-023-00424-4 (PMC10127322; doi:10.1186/s43058-023-00424-4)
Supplement: Supplementary file 1 — Additional file 1: Fig. 1. Data Extraction Guide for Universal Tumor Screening (UTS) Protocol Processes. Fig 2. Process and Contextual Differences within Organization 1. Fig 3. Initial Process Map for Organizational Unit 1A. Fig 4. Reconciled Process Map for Organizational Unit 1A. Fig. 5. Process Gap/Inefficiency to Optimization Component Conversion Table. Fig. 6. Matrix of UTS Protocol Optimization Levels by Organizational Unit. Fig. 7. Section of the CFIR Codebook Used for Qualitative Data Analysis. Fig. 8. Data Matrix of Factors Related to Intervention Characteristics from Organizational Unit 7. Fig. 9. Example of a Data Matrix Heat Map for Factors Related to Intervention Characteristics. Fig. 10. Example of Combined Codes for Factors Related to Intervention Characteristics. Fig. 11. Consolidated Data Matrix Heat Map for Characteristics of Intervention by Optimization Score. Fig. 12. Example of Collapsed Code ‘Evidence & Relative Advantage’ for all Organizational Units. Fig. 13. Final Heat Map of Factors Selected for Coincidence Analysis (CNA). [file 43058_2023_424_MOESM1_ESM.docx]

**Supplemental Materials**

**Step-by-step process mapping methodology and output directions and examples**

**Step 1:** **Code protocol descriptions from each stakeholder using a data extraction sheet to identify process steps**


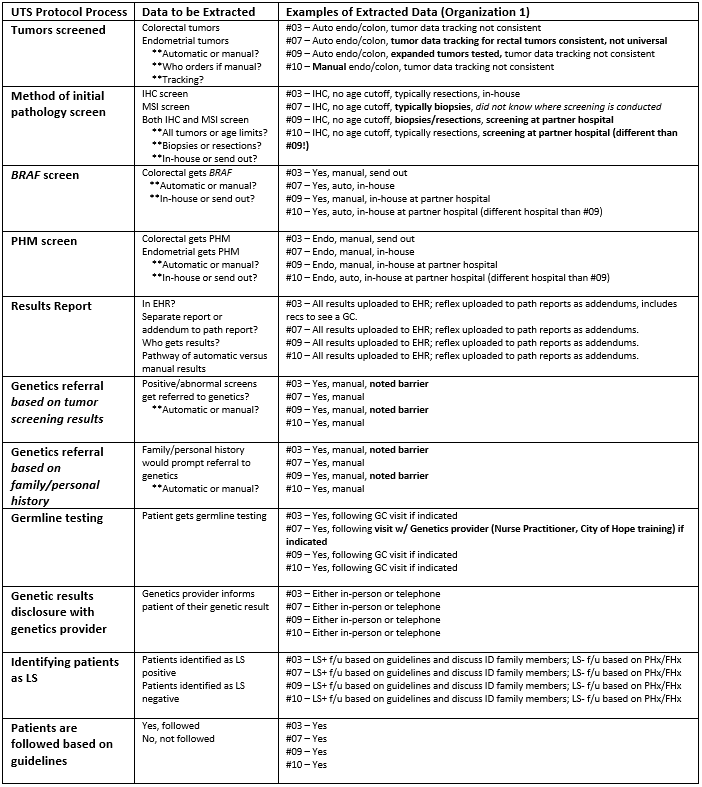
**Fig. 1 Data Extraction Guide for Universal Tumor Screening (UTS) Protocol Processes**

**Step 2:** **List process and contextual differences to define organizational units**

**Fig 2. Process and Contextual Differences within Organization 1**


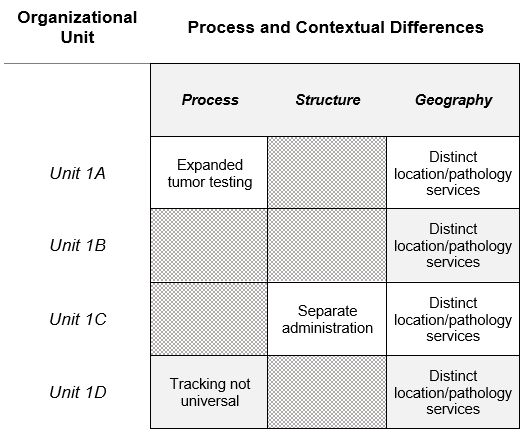


Fig. 2 Each organizational unit has its own configuration of process, structure, and geography. Organization 1 resulted in four organizational units due to this configuration. Other organizations ranged from one to six units.

**Fig 3.** **Initial Process Map for Organizational Unit 1A**

**Step 3:** **Create visual representations of protocols to document processes and stakeholder inconsistencies within each organizational unit**

*
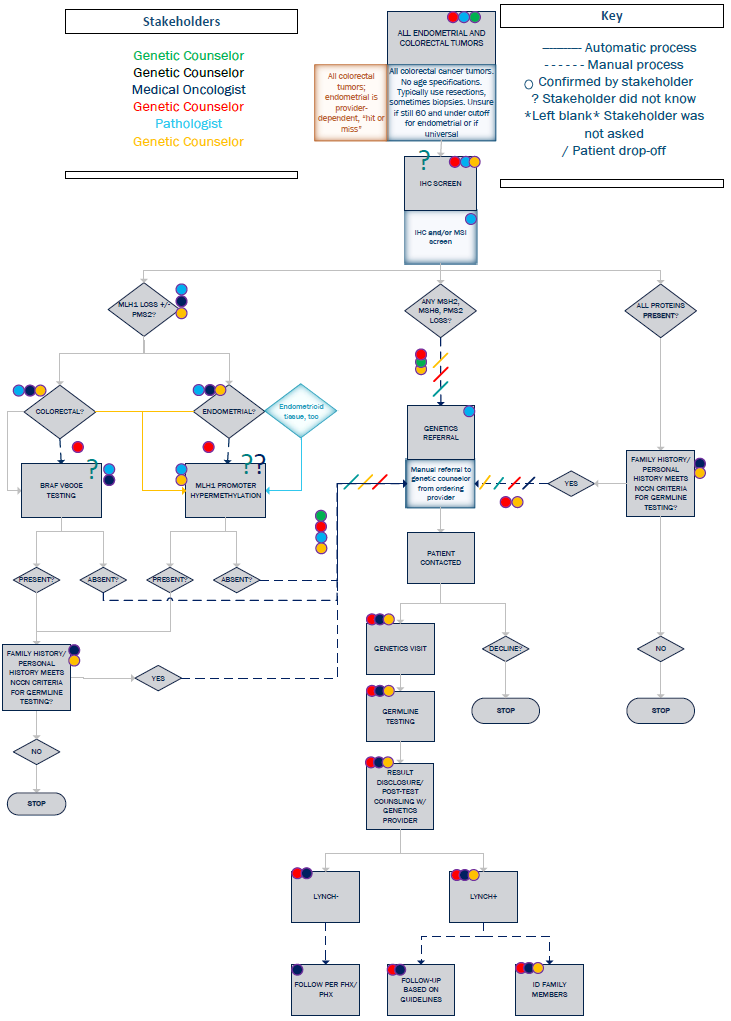
*

*Fig. 3* Key describes lines and symbols that were color-coded to represent varying stakeholder perspectives and to signify corroboration across stakeholders within an organization. Symbols were also used to identify and visually discern inconsistencies in stakeholder-reported interview data.

**Fig 4. Reconciled Process Map for Organizational Unit 1A**

**Step 4:** **Reconcile stakeholder inconsistencies to validate and finalize the process map for each organizational unit**


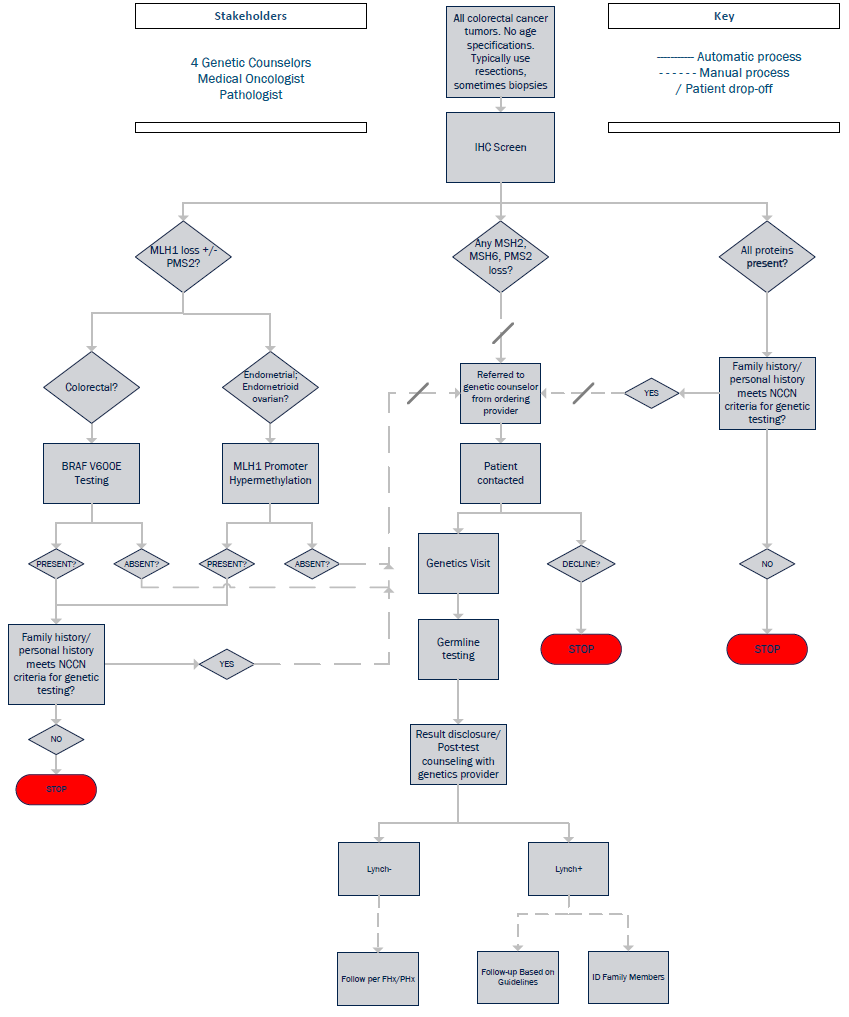


*Fig. 4* Completion of process mapping resulted in 19 reconciled process maps, one for each organizational unit.

**Step 5:** **Compare process maps across organizational units to find gaps and inefficiencies and define process optimization components**


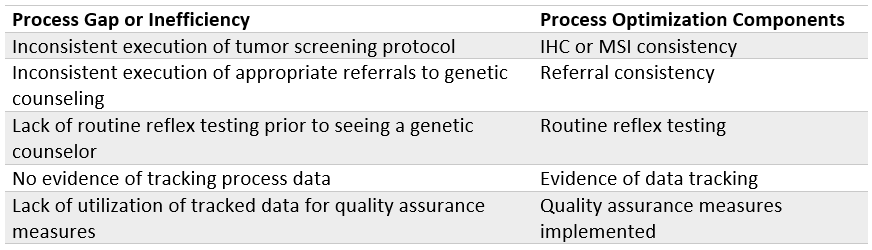
**Fig. 5 Process Gap/Inefficiency to Optimization Component Conversion Table**

**Step 6: Create overall scores representing optimization levels for each organizational unit**


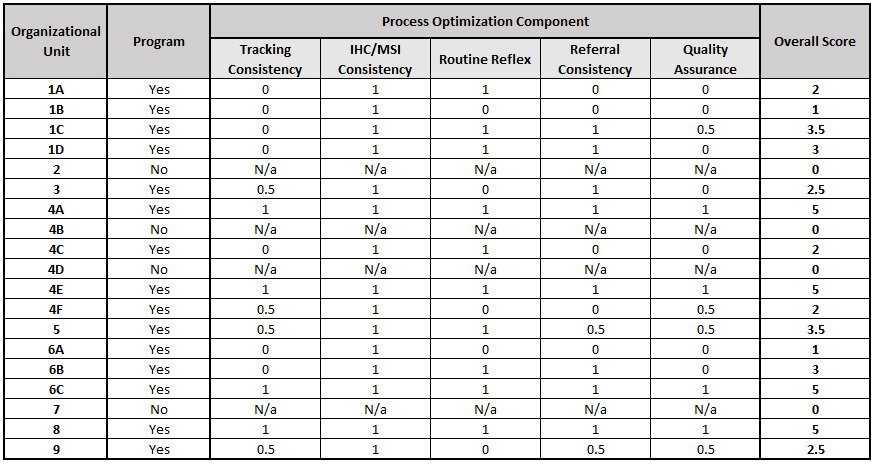
**Fig. 6 Matrix of UTS Protocol Optimization Levels by Organizational Unit**

*Fig. 6* Presence of process optimization component = “1”; Historical presence without current presence of optimization component, or unresolved discrepancy between stakeholders = “0.5”; Absence of process optimization component = “0”. Optimization components were not applicable (N/a) at organizational units without a program and received an overall optimization score of “0”.

**Step-by-step data matrix heat mapping methodology and output directions and examples**

**Step 1: Conduct formal qualitative analysis on stakeholder data**

**Fig. 7 Section of the CFIR Codebook Used for Qualitative Data Analysis**


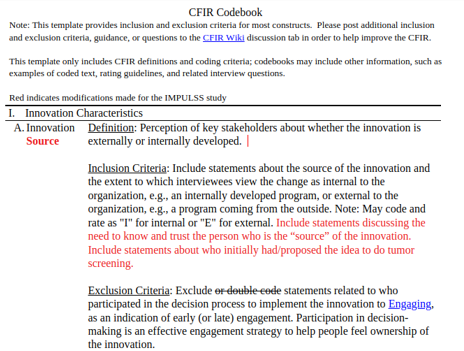


**Step 2: Create a data matrix for each organizational unit to compile and organize stakeholder data**


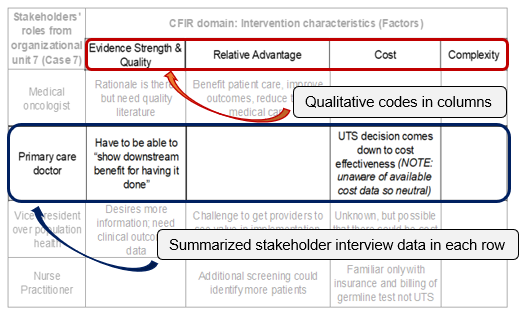
**Fig. 8 Data Matrix of Factors Related to Intervention Characteristics from Organizational Unit 7**

*Fig. 8* For simplicity, this data matrix represents interview data from a single organizational unit (Organizational Unit 7) for four factors.

**Step 3: Assign valences (color-coding) to begin heat mapping**

**Fig. 9 Example of a Data Matrix Heat Map for Factors Related to Intervention Characteristics**


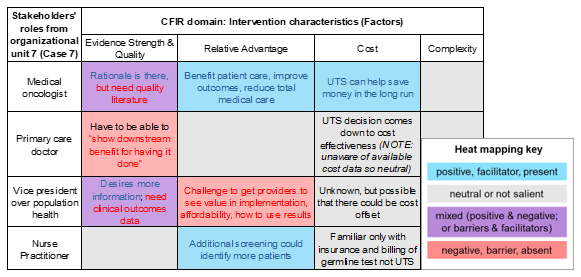


*Fig. 9* Colors were assigned solely to data points relevant to UTS implementation and/or maintenance. For simplicity, this data matrix heat map represents interview data from a single organizational unit (Organizational Unit 7) for four factors.

**Step 4: Combine stakeholder valences for each factor (column code) within organizational units**


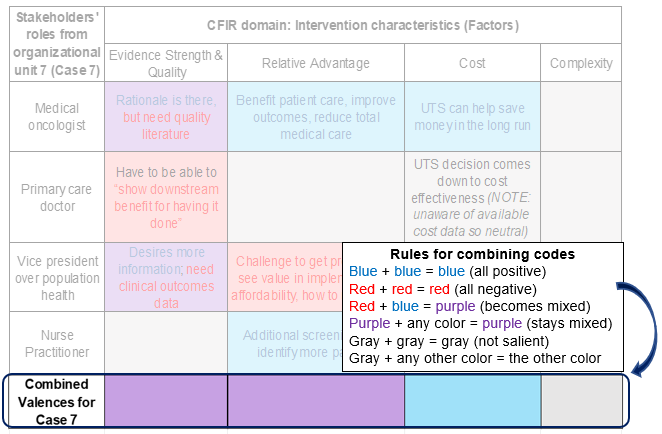
**Fig. 10 Example of Combined Codes for Factors Related to Intervention Characteristics**

**Step 5:** **Copy factor valences from each organizational unit and order units by the outcome (i.e., optimization scores that were determined during process mapping)**


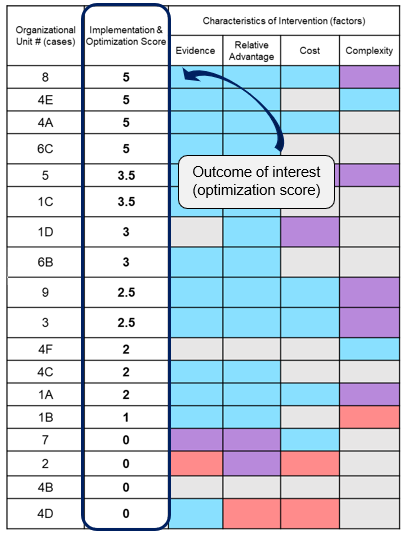
**Fig. 11 Consolidated Data Matrix Heat Map for Characteristics of Intervention by Optimization Score**

**Step 6: Collapse related or overlapping codes within factors for data reduction and simplification**


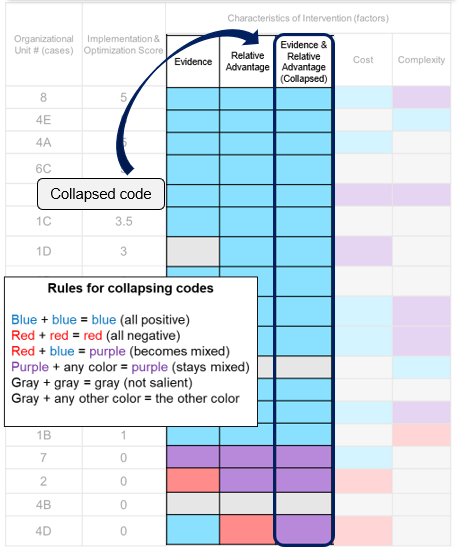
**Fig. 12 Example of Collapsed Code ‘Evidence & Relative Advantage’ for all Organizational Units**

*Fig. 12* Rules for collapsing codes were as follows: When factors that were combined all had positive valences, the valence remained positive and were color-coded blue; if all had negative valences the factor was negative (red); a mix of positive and negative valences resulted in the factor being assigned purple; a mixed valence with any other valence remained mixed (purple); when factors that were not salient or neutral were combined with each other they remained gray, but otherwise they took on the valence of the factor with which it was combined.

**Step 7: Compare data across organizational units and create a final data matrix heat map of selected factors as a preparatory step for future analysis**

**Fig. 13 Final Heat Map of Factors Selected for Coincidence Analysis (CNA)**


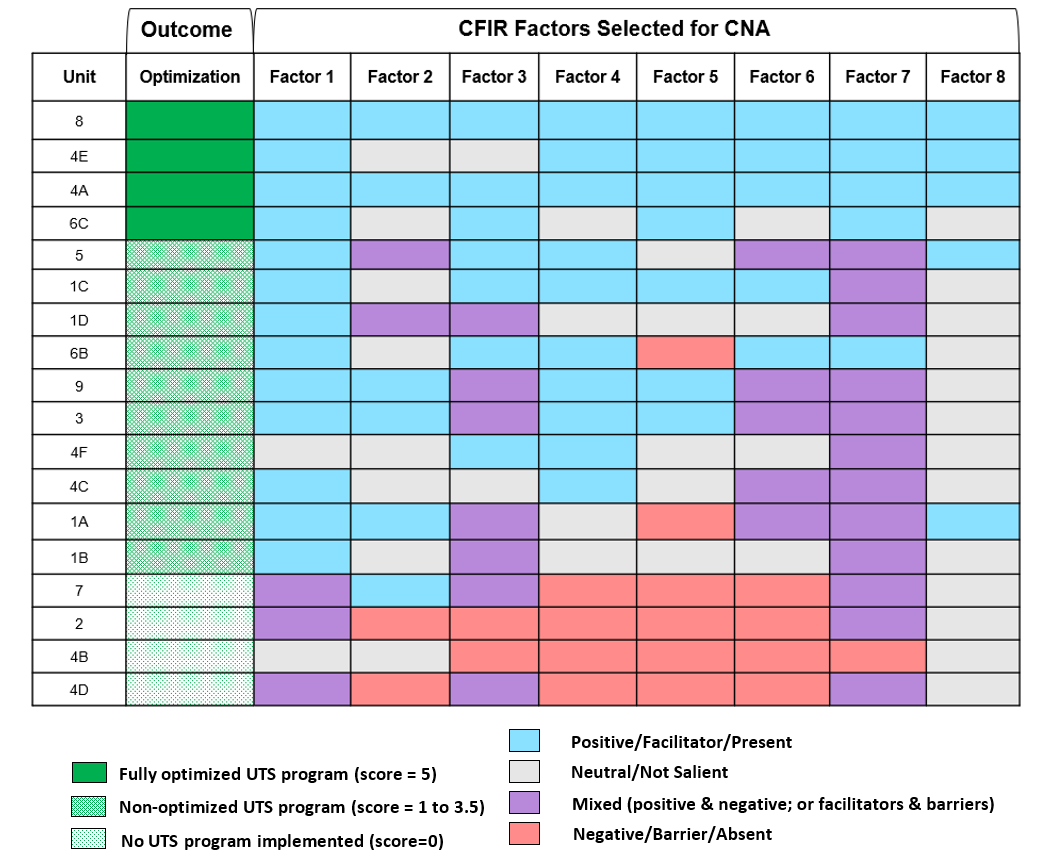


*Fig.13* Factor 1: Evidence & relative advantage; Factor 2: Cost; Factor 3: Knowledge & attitudes; Factor 4: Implementation champion; Factor 5: Maintenance champion; Factor 6: Planning & engaging stakeholders; Factor 7: Inner setting (except structural); Factor 8: Cosmopolitanism & peer pressure.
